# Mechanistic Insights into Bioprosthetic Heart Valve Calcification and Anti-Calcification Strategies

**DOI:** 10.31083/RCM36688

**Published:** 2025-05-20

**Authors:** Yuxiao Yang, Limin Lan, Yi Lin

**Affiliations:** ^1^Department of Clinical Medicine, School of Medicine, Xiamen University, 361102 Xiamen, Fujian, China; ^2^Fuzong Clinical Medical College of Fujian Medical University, 350025 Fuzhou, Fujian, China; ^3^Department of Cardiology, Dongfang Hospital of Xiamen University, School of Medicine, Xiamen University, 350025 Fuzhou, Fujian, China

**Keywords:** prosthetic heart valves, bioprosthetic valves, calcification

## Abstract

Prosthetic heart valves are crucial for treating valvular heart disease and serve as substitutes for native valves. Bioprosthetic heart valves (BHVs) are currently the most common type used in clinical practice. However, despite the long history of use, challenges remain in clinical applications, notably via valve calcification, which significantly affects longevity and quality. The mechanisms through which calcification occurs are complex and not yet completely understood. Therefore, this paper aims to provide a comprehensive review of developments in prosthetic valves, focusing on the calcification processes in bioprosthetic heart valves and the biological, chemical, and mechanical factors involved. In addition, we highlight various anti-calcification strategies currently applied to BHVs and assess whether anti-calcification approaches can prolong valve durability and improve patient prognosis. Finally, we describe the imaging methods presently used to monitor calcification clinically. Advances in nanotechnology and tissue engineering may provide better options for mitigating prosthetic heart valve calcification in the future.

## 1. Introduction

The prevalence of heart valve disease is increasing globally. According to the American Heart Association, the number of cases of valvular heart disease will increase to 24 million by 2024, underscoring the need for effective solutions [1]. Prosthetic heart valves have revolutionized the management of valvular heart disease and provided definitive therapy for patients with conditions such as aortic stenosis and mitral valve prolapse. These valves act as important backups when natural valves are unable to provide adequate blood flow because of calcification, stenosis or regurgitation [2]. Despite the success of valve replacement surgeries, clinical complications still persist, whereby calcification is one of the worst, affecting both the durability of the valve and patient outcomes [3]. The calcification of prosthetic valves leads to their stiffness and malfunction, which may require further intervention [4, 5, 6, 7]. Thus, valve calcification is considered to be one of the important causes and manifestations of structural valve degeneration (SVD). However, valve calcification is influenced by a variety of factors, such as valve type, source, treatment process, underlying diseases, genes, metabolism, etc. Although new valve replacements continue to be researched and developed as technology advances, bioprosthetic valves remain the most commonly used type of prosthetic heart valve today. As calcification is an important factor in valve longevity, it is very important to explore the relevant mechanisms and seek targeted anti-calcification strategies. In addition, detecting calcification early and monitoring the calcification process are also clinical problems. Therefore, we sought to review and analyze these issues in a systematic manner.

## 2. Current Types of Prosthetic Heart Valves

**Fig. 1.**
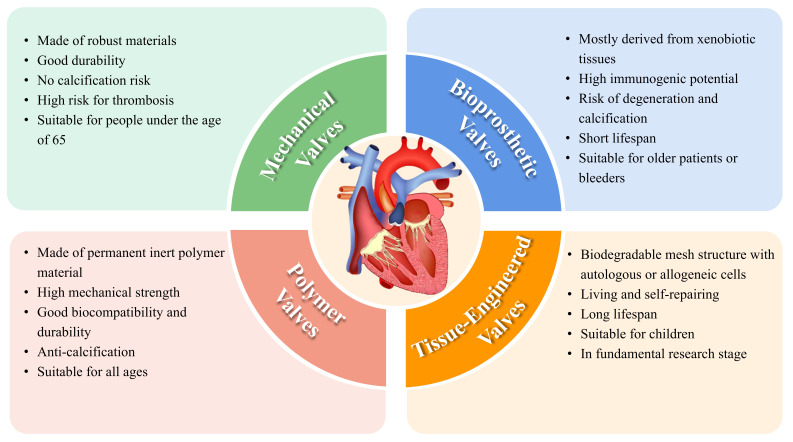
**Overview of current prosthetic heart valves**.

Prosthetic heart valve manufacturing methods are categorized into nonbiological and biological methods. Nonbiological valves are valves without living cell/tissue elements, such as polymer valves, bioprosthetic valves, and mechanical valves. Biological methods aim to replicate native heart valves by combining living cells (valve cells, stem cells) with biocompatible scaffolds (biopolymers, cell-generated extracellular matrix, and synthetic polymers). In clinical settings, mechanical and bioprosthetic valves are the most commonly used types of prosthetic heart valves. With advances in materials science, tissue-engineered and polymer valves are attracting increasing attention. However, each type has advantages and disadvantages. A summary of the classification of prosthetic heart valves is depicted in Fig. 1.

### 2.1 Mechanical Valves

Mechanical heart valves (MHVs) have been in use for more than 50 years. The advantage of MHVs is their durability, since these valves are made from materials such as pyrolytic carbon, titanium and other metallic alloys, and they last for 20 years or more [8, 9]. They have long-term stability, which minimizes the risk of reoperation, which is especially important for young patients or those who have a longer life expectancy. Individuals with mechanical valves, on the other hand, require lifelong anticoagulation therapy because these valves increase the risk for blood clot formation. Moreover, this anticoagulation requirement is associated with increased bleeding hazard, making these valves less suitable for patients with bleeding disorders or those who cannot strictly follow anticoagulation protocols [8, 9, 10].

Recent developments have improved the hemodynamic characteristics and biocompatibility of mechanical valves, and advancements in valve structures, such as bileaflet and tilting-disc models, have enhanced flow characteristics and decreased turbulence, thus lowering thrombogenicity and structural deterioration [11, 12, 13]. Scientists are also working on the development of composite valves that are made of both metals and polymers to mimic the natural movement of native valves and eliminate the need for anticoagulants [12, 14, 15].

### 2.2 Bioprosthetic Valves

Bioprosthetic heart valves (BHVs) are categorized by source: autografts (from the patient’s own tissue, for example, the pulmonary artery), allografts (from donors) and xenografts. Owing to limitations such as material sourcing, ethical considerations and complications, the use of autograft and allograft valve transplants remains restricted. Clinically, xenograft valves are the primary type of BHV, and most research improvements have focused on these valves. Xenogeneic BHVs are sourced from animal tissues, usually bovine pericardium or porcine aortic valve tissue, and are treated with cross-linking agents such as glutaraldehyde (GLUT) to increase the sturdiness of the tissue [16, 17, 18]. These valves exhibit more physiological characteristics of the native valves and are not usually associated with long-term anticoagulation therapy, making them ideal for elderly patients or those at risk for bleeding. However, BHVs exhibit reduced durability compared with mechanical valves primarily because of calcification and structural degradation over time [3].

To address these durability issues, new biomaterials, such as decellularized fish bladder tissue, have been developed. Fish bladder tissue has a natural collagen matrix with anti-calcification properties that can improve the biocompatibility of the valve and potentially increase its durability [19, 20, 21]. Research is also being conducted on enhanced cross-linking strategies that minimize immune reactions while maintaining the mechanical characteristics of the valve [21]. Nevertheless, the problem of calcification of bioprosthetic valves still persists and results in lower durability compared to mechanical valves.

### 2.3 Polymer Valves

Polymer heart valves (PHVs) provide excellent mechanical strength and fatigue resistance, along with the required flexibility, biostability and durability. PHVs can theoretically be implanted in patients of any age. Polymer valves are available in a wide range of biocompatible and biostable polymers. These valves are superior to biologic valves in terms of freedom from antigens (e.g., galactose α-1,3-galactose and N-acetylneuraminic acid) [2]. The earliest PHV materials include polysiloxanes, polytetrafluoroethylene, and polyurethane, but these materials do not effectively prevent prosthetic degeneration and complications given limitations in their chemical properties and surface structure. With improvements in polymer manufacturing methods and advances in nanotechnology, new polymer materials, such as polyhedral oligomeric silsesquioxane poly (carbonate-urea) urethane (POSS-PCU), nanocomposite based on the functionalized graphene oxide and poly (carbonate-urea) urethane (FGO-PCU, Hastalex), nanocomposite polyvinyl alcohol (PVA) and bacterial cellulose (PVA-BC), exhibit improved mechanical properties and biocompatibility [15]. Some valves based on new polymer materials have already been used in *in vitro* and animal studies as well as clinical trials and have shown promising results.

### 2.4 Tissue-Engineered Valves

Tissue-engineered heart valves (TEHVs) constitute the next generation of heart valve prosthetics and are designed to develop living, self-repairing heart valves through tissue engineering. These valves employ biodegradable meshes populated with autologous or allogeneic cells that, in due course, form new valve tissue [22, 23, 24]. TEHVs have the potential to transform valve replacement therapy since the valve can grow and change in size in the body, especially for children [25, 26, 27].

TEHV scaffolds are generally categorized into acellular and synthetic scaffolds. Acellular scaffolds are obtained from decellularized human or animal tissues and have a structure that mimics the native valve structure [28, 29]. Scaffolds made from synthetic materials, including biodegradable polymers, allow for the fine-tuning of mechanical characteristics and degradation profiles [14, 30]. Nevertheless, TEHV has several limitations, including scaffold degradation, calcification, and cell incorporation [22, 31]. Current research is being conducted to optimize scaffold materials and promote endothelialization to obtain better long-term results. Notably, despite the universal appeal of TEHV, the technique has not been used clinically.

## 3. Mechanism of Calcification in Natural Heart Valves

The calcification of heart valves is a dynamic process that is determined by biochemical, genetic, and mechanical factors. Although age is the most common cause of degeneration, other factors, such as endothelial cell dysfunction, lipid accumulation, and immune reactions, contribute to calcification.

### 3.1 Endothelial Cell Damage

The endothelial layer is the first barrier to calcification [32]. Shear forces and mechanical stress on the valve surface can disrupt endothelial cells and make the underlying tissue susceptible to infiltration by lipids and immune cells [33, 34, 35]. When endothelial cells are damaged, they are no longer able to prevent clot formation and become proinflammatory, promoting calcification [36]. Damaged endothelial cells release adhesion molecules that allow immune cells to attach to the tissue, increasing inflammation and accelerating calcification [37, 38].

### 3.2 Lipid Infiltration

Lipid accumulation, especially low-density lipoprotein (LDL) accumulation, is involved in the process of calcification of natural valves [39, 40]. When LDL is oxidized, it forms ox-LDL, which causes inflammation that attracts macrophages, and these macrophages take up ox-LDL and become foam cells, which cause atherosclerosis-like lesions on the valve [41]. Foam cells secrete cytokines and growth factors that induce the osteoblastic phenotype of valve interstitial cells (VICs) and lead to mineralization of the valve matrix [42]. Lipids may also be involved in amyloid deposition in valve calcification through the formation of amyloids from misfolded apolipoproteins, thereby altering ion concentrations, providing templates for mineral deposition, and promoting apoptosis in valve interstitial cells [43].

### 3.3 Immune Response

The involvement of the immune system in calcification is now well appreciated, with T cells and macrophages representing key players in the inflammatory process that leads to calcification [37, 44]. Immune cells secrete matrix metalloproteinases (MMPs) and proinflammatory cytokines that degrade the extracellular matrix (ECM) and promote calcification [44, 45]. In addition, immune cells release cytokines that stimulate osteogenic processes in VICs. As a result, calcium nodules are formed, and the valve becomes hardened [41, 46].

## 4. Calcification Mechanisms in Bioprosthetic Valves

BHV calcification is similar to that of natural heart valves because of its functional consistency (unidirectional blood flow control) and structural similarity. Shear stress, lipid deposition (especially LDL), and endothelial damage trigger inflammatory responses and immune cell infiltration, leading to cytokine production, neoangiogenesis, osteoblast formation, and calcification [47]. LDL deposited in valve tissue oxidizes to ox-LDL, is phagocytosed by macrophages to form foam cells, and stimulates osteogenic inflammation [42, 48]. Additionally, because BHVs are foreign materials, they inherently differ from natural heart valves and contain xenogeneic antigens. BHVs require preimplantation processing (e.g., decellularization and cross-linking), influencing their calcification mechanisms.

### 4.1 Alloimmune Response

In addition to the immune inflammation, cell infiltration, and cytokine secretion observed in natural valve calcification, nonspecific plasma protein adsorption can activate the complement system, platelets, coagulation cascade, and cell adhesion [49, 50]. Infiltrating immune cells release proteases, degrading the ECM, which contributes to calcification by releasing calcium ions and providing binding sites [51]. In particular, the xenoantigens galactose-α1,3-galactose (α-Gal) and N-glycolylneuraminic acid (Neu5Gc) are thought to be important in triggering the immune response to mediate calcification [52]. Anti-Gal antibodies are the most abundant natural antibodies in humans and constitute the main immune barrier in xenotransplantation [53]. Anti-Gal in human circulation binds to α-gal epitopes on the endothelial cells of xenografts and induces complement-mediated cytolysis, followed by platelet aggregation, small-vessel occlusion, vascular bed collapse, and hyperacute rejection of the xenografts [54]. Currently, *Gal* knockout (KO) pigs created using gene knockout are better able to avoid hyperacute rejection. However, the problem of calcification due to immunogenicity is not completely resolved in this model given the presence of other immune epitopes. Neu5Gc is another key xenoantigen found primarily in glycoproteins and gangliosides in most mammals, but humans do not synthesize Neu5Gc. In humans, Neu5Gc obtained through dietary intake induces natural immunity and produces anti-Neu5Gc antibodies in the serum [55]. Antibody-antigen binding promotes valve calcification, and the levels of Neu5Gc, anti-Neu5Gc immunoglobulin G (IgG), and complement deposition are much greater in calcified BHVs compared with calcified natural aortic valves [52]. In addition to the two important antigenic epitopes described above, other antigenic epitopes, such as Sda, may also participate in the process of valvular calcification, and these antigenic species and the safety of methods of elimination need to be revealed by further research.

### 4.2 Decellularization Impact

Decellularization reduces BHV immunogenicity by removing cells and nucleic acids from the ECM using physical (freeze-thaw, high hydrostatic pressure, supercritical fluid) and chemical methods (surfactants, acids, bases, and enzymes such as trypsin) [56]. Physical methods preserve ECM integrity but are less effective immunogenically. Chemical methods are widely used but may damage ECM proteins [57]. Combination methods optimize results, but residual immunogenicity and ECM changes can affect immune responses and calcification. Some *in vitro* experiments have shown a higher rate of calcification in decellularized porcine aortic valves than in those fixed with GLUT, and this difference may be attributed to tissue surface modification and residual cellular debris during decellularization [58].

### 4.3 Cross-Linking Impact

Natural collagen undergoes intramolecular and intermolecular crosslinking via enzymatic processes or nonspecific glucose interactions, resulting in the formation of advanced glycation end products. These crosslinks protect proteins from degradation and maintain their stability [59]. BHV cross-linking aims to enhance these crosslinks using physical and chemical techniques, influencing calcification.

Currently, GLUT is the most commonly used cross-linking agent because of its highwater solubility, fast reaction rate, superior cross-linking properties, and cost effectiveness. However, GLUT cross-linking can induce valve calcification through several mechanisms. For example, GLUT treatment causes cell death, stops membrane ion pumps, increases the level of intracellular calcium, and promotes nucleation and calcification [3]. Glycosaminoglycans in the ECM are not cross-linked by GLUT, leading to degradation under mechanical stress or proteases, exposing calcification-prone areas and facilitating collagen mineralization [3]. Unlike collagen, GLUT cannot stabilize elastin because of insufficient active amino groups, making elastin susceptible to mechanical and enzymatic degradation, resulting in calcification [60]. GLUTs form polymers through aldol condensation in water, with free aldehyde groups persisting and causing cytotoxicity [61]. The study has shown that aldehyde content is correlated with increased tissue calcium levels [62]. GLUT cross-linked biomaterials carry a negative charge, attracting positively charged calcium ions from host plasma and leading to calcification [61].

### 4.4 Valve Implantation Method

After decades of development, transcatheter aortic valve replacement (TAVR) has demonstrated the feasibility of transcatheter interventions for the treatment of heart valve disease. The bioprosthetic valves used in TAVR need to be folded and then unfolded after being accessed through a catheter in the appropriate position. The impact of different procedures on future valve calcification remains inconclusive. However, TAVR may lead to calcification or even SVD due to the use of a thinner pericardium and biomaterial microinjury during the curling process. The Nordic Aortic Valve Intervention (NOTION) trial randomized patients at low surgical risk for TAVR or surgical aortic valve replacement (SAVR) and reported their 10-year clinical and bioprosthesis prognoses, revealing that the risk of severe bioprosthesis SVD after TAVR was lower than that of SVD after TAVR. Compared with SAVR, prosthesis SVD after TAVR has a lower risk [63]. Another retrospective study reported similar findings [64]. However, to varying degrees, the aforementioned studies involved small sample sizes, survival bias, and multiple influencing factors, and the relationship between implanted valve calcification and the surgical approach needs to be further investigated.

## 5. Genetic Factors Influencing Calcification in Prosthetic Heart Valves

Recent studies have shown that genetic predispositions significantly influence calcification in both natural and prosthetic heart valves [65, 66]. Specific genes related to osteogenesis, the immune response, and lipid metabolism are associated with increased calcification risk, which impacts patient outcomes and the longevity of bioprosthetic valves. Knowledge of these genetic factors provides the possibility of developing specific treatments for calcification in genetically predisposed patients.

### 5.1 Key Genetic Pathways in Calcification

Multiple genes involved in the process of calcification in the cardiovascular system, such as Runt-related transcription factor 2 (*RUNX2*), are essential for osteogenic differentiation and can cause calcification when overexpressed [67]. Patients with *RUNX2* gene mutations may undergo more severe calcification in both bioprosthetic valves and native heart valves, and this gene is normally active in bone formation but can be abnormally switched on in heart tissue, leading to mineralization [68].

Another important signaling pathway is the bone morphogenetic protein (BMP) pathway, in which BMPs, especially *BMP-2*, are known to be strong promoters of osteogenic differentiation in vascular tissues [69]. Alleles that increase BMP expression are involved in the development of calcific aortic stenosis and may promote calcification of prosthetic valves [70]. In addition, mutations in SMAD (homolog of *Caenorhabditis elegans* Sma and the *Drosophila* mad, mothers against decapentaplegic) proteins that are involved in BMP signaling are associated with abnormal mineralization and how the body controls calcification responses in implanted valves [71].

### 5.2 Inflammation and Immune Response Genes

The immune response to implanted valves is one of the major contributors to calcification, especially in xenogeneic bioprosthetic valves, in which polymorphisms in proinflammatory cytokines, including tumor necrosis factor alpha (TNF-α), interleukin-1 beta (IL-1β), and interleukin-6 (IL-6), are associated with an enhanced immune response leading to local inflammation and calcification [72, 73]. These cytokines activate macrophages, which subsequently create osteogenic precursor cells within the valve tissue, a process that increases calcification.

### 5.3 Lipid Metabolism and Genetic Predisposition

Genes involved in lipid metabolism are also involved in calcification, mainly through the oxidation of LDL, such as the apolipoprotein E (*APOE*) gene, which is involved in lipid transport and metabolism in the body. The *APOE ε4* allele increases the risk of LDL oxidation in patients, which leads to inflammatory reactions in valve tissues and calcification, and oxidized LDLs penetrate the valve scaffold, activate macrophages, and promote the osteoblastic transformation of VICs [74, 75].

## 6. Metabolic Factors Contributing to Calcification in Heart Valves

Diabetes, chronic kidney disease (CKD), and hyperlipidemia are metabolic disorders that increase the risk of calcification in prosthetic heart valves. These conditions affect mineral handling and inflammatory processes in the body and promote the calcification and degradation of bioprosthetic valves.

### 6.1 Hypercalcemia and Phosphate Dysregulation

Hyperphosphatemia is a common feature in patients with CKD and contributes to vascular and valvular calcification, as phosphate combines with calcium to form hydroxyapatite crystals that precipitate in valve tissues. This condition encourages calcification through osteogenic signaling pathways, including the activation of genes that are involved in bone formation, such as alkaline phosphatase (ALP) and osteopontin [76, 77].

Furthermore, CKD patients have disordered calcium metabolism, and high serum calcium leads to mineral deposition on the valve surface. Phosphate binders, commonly prescribed to manage hyperphosphatemia, can worsen these problems by increasing serum calcium concentrations and thereby increasing the risk of calcification [76, 78].

### 6.2 Diabetes Mellitus and Advanced Glycation End Products (AGEs)

Diabetes mellitus is associated with increased calcification risk, primarily because of the formation of AGEs at high glucose concentration [79]. AGEs are proteins or lipids that are glycated because of high blood sugar levels and cause tissue hardening and calcification, including prosthetic valves, and they interact with receptors on immune cells, especially macrophages, to stimulate inflammatory signaling that leads to calcification [71, 80]. AGEs alter the mechanical properties of valve tissues by cross-linking collagen and increase the susceptibility of valves to calcification [81]. A literature review revealed that diabetic patients undergo calcification of bioprosthetic valves at a faster rate than nondiabetic patients do; thus, they require reoperations more frequently [80]. AGE inhibitors are among the preventive measures that are being researched for their ability to slow calcification in diabetic patients.

### 6.3 Dyslipidemia and Calcification

Dyslipidemia, which is defined by increased LDL and triglyceride levels, is involved in prosthetic valve calcification through lipid infiltration and oxidation. As discussed in Section 3.2, oxidized lipids activate inflammatory processes and attract immune cells to the valve site that subsequently become foam cells. These cells enhance the differentiation of VICs into osteoblast-like cells and increase the rate of mineralization [41]. In the case of statins, which are used to treat dyslipidemia, the effects on calcification have been inconclusive. However, newer drugs in the lipid-lowering family, such as proprotein convertase subtilisin/kexin type 9 (PCSK9) inhibitors, may provide benefits in preventing lipid-induced calcification [82].

### 6.4 Impact of Hormonal and Mineral Imbalances

Calcification is also regulated by hormonal factors, especially parathyroid hormone (PTH). PTH is involved in calcium and phosphate balance, and hyperparathyroidism may cause an excess of calcium phosphate, which increases the calcification potential [83, 84]. Dietary and pharmacological interventions may help reduce calcification risk in high-risk populations, but more research is needed to fine-tune these strategies.

## 7. Anti-Calcification Strategies

Current strategies to address artificial heart valve calcification focus on its mechanisms and are categorized into systemic and local approaches. Systemic anti-calcification strategies aim to reduce or eliminate systemic risk factors linked to valve calcification, primarily through pharmacological treatments. Local strategies involve special treatments or improvements to artificial valves to enhance their physical and chemical properties, thereby reducing or preventing calcification (Table 1).

**Table 1.** **Anti-calcification strategies of bioprosthetic heart valves**.

Category	Aim	Specific methods
Systemic strategies	Applying drug therapies to reduce calcification risk	Stain
		Immunosuppressants
		Management of underlying disease
Local strategies	(i) Target the physical and chemical properties of valve materials to resist calcification	Advanced decellularization techniques
		Crossing-linking innovations
		Adding coatings to BHV surface
	(ii) Maintain biocompatibility and mechanical integrity	Elimination of xenoantigens
		Polyphenol-based treatment
	(iii) Improve long-term performance and durability	Dry valve techniques

BHV, bioprosthetic heart valve.

### 7.1 Systemic Anti-Calcification Strategies

#### 7.1.1 Statin Therapy

Statins work by competitively inhibiting 3-hydroxy-3-methylglutaryl-coenzyme A (HMG-CoA) reductase, reducing endogenous cholesterol synthesis, which increases LDL receptor activity and lowers total cholesterol and LDL levels [85]. They also reduce triglycerides and increase high-density lipoprotein (HDL). Lowering plasma lipid levels may help prevent artificial heart valve calcification given the role of lipids in calcification. A study showed that inactivating the *mttp* gene in hypercholesterolemic mice normalized oxidative stress and reduced pathogenic signaling, preventing aortic valve disease progression [86]. Other studies have indicated that statins (e.g., rosuvastatin and atorvastatin) reduce BHV calcification by lowering IL-6 and BMP levels [87, 88]. However, the role of statins in valve calcification is debated. Kulik and colleagues [89] reported that lipid-lowering therapies did not delay calcification postaortic valve replacement. A meta-analysis reported no impact on valve structure, function, calcification, or clinical outcomes, despite cholesterol-lowering effects [90]. Discrepancies may arise from differences between animal models and humans and from varying study methods. Although statins might be used to treat or delay valve calcification, high-level evidence is needed. The nonlipid effects of statins, such as improving endothelial function, resisting oxidation and reducing inflammation, could also be beneficial in treating valve calcification.

#### 7.1.2 Immunosuppressive Drug Therapy

Research indicates a reduction in calcific degeneration of the valves in BHV transplant patients with aortitis who receive steroid treatment [91]. Therefore, given the role of immune responses in artificial heart valve calcification, the use of immunosuppressive drugs could also represent a potential therapeutic approach. However, the systemic use of immunosuppressive drugs may produce severe side effects, such as an increased risk of infections. Currently, some researchers are focusing on the use of surface-modified nanoparticles that bind to specific receptors that are overexpressed in atherosclerosis to achieve precise and efficient therapeutic effects, thereby reducing adverse impacts on nontargeted tissues [92, 93]. A similar approach might be applied to immunotherapy for treating heart valve calcification.

#### 7.1.3 Management of Underlying Diseases

As previously noted, diabetes, kidney disease, and hormonal imbalances can lead to heart valve calcification. Treating these conditions may help reduce calcification. However, some treatments may worsen it. For example, calcium-based phosphate binders, which are used for hyperphosphatemia in chronic kidney disease, can increase cardiovascular risk [94]. Conversely, sevelamer hydrochloride, an alternative to calcium-based phosphate binders, was found to reduce BHV calcification through calcium‒phosphate regulation and anti-inflammatory effects independent of these elements [95, 96].

### 7.2 Local Anti-Calcification Strategies

Currently, local strategies for preventing calcification primarily target the physical and chemical properties of the valve materials themselves, aiming to create prosthetic valves that are resistant to calcification while maintaining biocompatibility and mechanical integrity. These strategies include improving the decellularization process, improving cross-linking chemistry, eliminating xenoantigens, adding coatings to the valve surface, polyphenol-based treatment and drying biological valve techniques.

#### 7.2.1 Enhancing Decellularization

Two of the most important steps in the preparation of bioprosthetic valves are decellularization and cross-linking because they affect the calcification resistance of the valve. New strategies are designed to enhance these techniques to increase calcification resistance without compromising tissue properties. Decellularization is a critical step in reducing the immunogenicity of BHVs because it eliminates cell components that may cause an immune response after implantation [29, 97, 98]. New agents and methods are being developed to enhance the process of antigen removal while maintaining ECM integrity. Heat, ultrasound, and pressure are not very effective when used individually but are very effective when used in combination with chemical treatments. Vacuum-assisted decellularization improves efficiency and reduces time without affecting valve properties, possibly by enhancing chemical distribution [99, 100]. Researchers suggest incorporating this parameter in the design of decellularization protocols [99].

Chemical decellularization agents, such as detergents and enzymes, offer another level of precision but have potential trade-offs [101, 102]. Detergents are categorized as ionic detergents, nonionic detergents, and zwitterionic detergents [103]. Sodium dodecyl sulfate (SDS), for example, is highly effective at dissolving cellular structures (such as cell and nuclear membranes) and removing antigenic cellular components, but it can damage ECM proteins if it is used at high concentrations or for extended durations, causing degeneration [104, 105]. Researchers are refining protocols by using submicellar concentrations of SDS or combining detergents with shorter wash times, balancing cell removal with ECM preservation [106, 107]. Controlling detergent residues to approximately 50 ng/mg may minimize toxic effects and will not impair subsequent endothelial cell functions [108]. Triton X-100 is a representative nonionic detergent widely used in various decellularization protocols. It targets lipid-lipid and lipid-protein chemical bonds without disrupting protein-protein interactions, effectively preserving the collagen structure of the ECM and maintaining its mechanical and biochemical properties [109, 110]. However, Triton X-100 is typically not used to decellularize tissues rich in glycosaminoglycans because it is less effective at removing antigenic components; this has led to explorations of combining nonionic and ionic detergents to achieve optimal results [101, 111].

Enzymatic methods are also common for decellularization and can effectively remove cell debris and other undesirable ECM components. Trypsin is a frequently used enzyme. However, prolonged exposure can lead to reductions in elastin and glycosaminoglycan (GAG) contents within the ECM [98]. The study has shown that exposure to 0.05% trypsin for 24 hours can cause irreparable damage to the ECM [112]. Pepsin is another commonly used enzyme. Additionally, different enzymes, such as α-galactosidase, can be selected on the basis of the specific tissue components to be removed to eliminate α-Gal xenogeneic epitopes, thereby reducing tissue immunogenicity [113]. To obtain the best decellularization, reduce immunogenicity and maintain the ECM structure to the maximum extent possible, it is necessary to consider the type of enzyme, its concentration, and the duration of the treatment.

#### 7.2.2 Improving Cross-Linking

BHV tissues are often cross-linked with GLUTs to increase their stability, but GLUT residues contain aldehyde groups that bind calcium ions, leading to calcification [47, 114, 115]. To this end, researchers have developed methods to eliminate these aldehyde residues. For example, when GLUT-fixed tissues are exposed to agents such as adipic acid diacyl hydrazide (AADH) or glutathione, free aldehyde groups are blocked, and calcification resistance and inflammation are improved [114, 115]. Some of the new cross-linking agents under consideration as GLUT substitutes include dialdehyde chondroitin sulfate and formaldehyde xanthan gum, and these new cross-linkers provide better stability of the ECM while exhibiting improved resistance to calcification [114, 115].

In addition to modifications based on GLUT cross-linking, researchers are constantly developing new cross-linkers to circumvent the major limitations of GLUT cross-linking. Research has demonstrated that double cross-linking methods that employ zwitterionic copolymers can form stable covalent linkages within the ECM without eliciting aldehyde-related cytotoxicity that leads to calcification [114]. A study of secondary cross-linking of bovine pericardium using oxidized chondroitin sulfate and an amphoteric radical copolymerization system instead of GLUT demonstrated that the products presented desirable mechanical properties and anti-calcification, anti-coagulant, and anti-inflammatory abilities in *in vivo* and *in vitro* experiments [116]. The GLUT-crosslinked BHVs modified with the robust polyvinyl alcohol-based hydrogel embedded with recombinant humanized collagen type III and tannic acid were shown to possess long-term anticoagulant activity, accelerated endothelialization, a mild inflammatory response and anti-calcification properties [117]. Synergistic cross-linking of porcine pericardium with dialdehyde xanthan gum and curcumin also resulted in better anti-calcification and anti-inflammatory ability compared with GLUT cross-linking in *in vitro* experiments [118]. Although these novel cross-linkers show ideal prospects for anti-calcification, more conclusive evidence is still needed to support the effectiveness, safety and even economy of their long-term effects if they are to replace GLUTs as widely used cross-linkers. In addition, these new cross-linkers also suffer from the problems of complex reactions, high catalyst limitations, and easy contamination of samples by byproducts to varying degrees.

#### 7.2.3 Adding Coating to Surfaces

The addition of coatings to the surface of BHVs is also a proven anti-calcification method. With advances in materials science, polymers show considerable potential in this area. Researchers grafted poly(2-methoxyethyl acrylate) (PMEA) onto porcine pericardium (PP) pretreated with GLUT and methacrylate polylysine to fabricate a PMEA-coated porcine pericardium, and the results demonstrated that the PMEA coatings significantly reduced PP calcification [119]. Luo *et al*. [120] coated a hybrid hydrogel of sulfobetaine methacrylate and methacrylate hyaluronic acid onto the surface of decellularized heart valves modified with methacrylic anhydride and then grafted the endothelium-affinity peptide, which showed better anti-calcification and endothelialization potential than BHV cross-linked with GLUT. Porcine pericardium cross-linked with bromo bicyclic-oxazolidine (OX-Br) instead of GLUT exhibits good resistance to calcification, endothelialization, thrombosis and infection in polymer brush-grafted BHV material [121]. Nanotechnology has also been used in this field. Guldner *et al*. [122] added a 30-nm-thick titanium nanocoating to GLUT-fixed bovine pericardium, which better avoided calcification of heart valves using a mechanism whereby the titanium nanocoating reduces immune complex deposition and immune cell adhesion to valvular collagen and physically blocks the grafted valves from contacting various known and unknown antigenic epitopes on the valve with blood. The surface structure of the coating also has an optimal endothelialization capacity, which ensures a certain degree of long-term anti-calcification properties.

#### 7.2.4 Elimination of Xenoantigens

As mentioned earlier, the immunogenicity of allograft valves and the immune response after implantation are important mechanisms that may lead to valve calcification. Among these, the α-Gal antigen is one of the most important and has received the most attention in recent years. There is evidence that the implantation of bioprosthesis induces persistent α-Gal-specific IgG immunoreactivity in valve recipients in an age-dependent manner [123, 124]. It can be assumed that the elimination of α-Gal epitopes in the grafts is very beneficial for the anti-calcification and life extension of BHV. One study showed that the treatment of porcine heart valves and pericardial tissue with α-galactosidase effectively removed α-galactose epitopes without affecting the biomechanical properties of the tissue [125]. Naso* et al*. [126] confirmed that the current GLUT treatment routinely performed on BHV inactivates only approximately half of the α-Gal epitopes. Based on these findings, the researchers developed a novel treatment called FACTA, where the tissue was incubated in an isotonic solution consisting of a highly selected mixture of food-grade molecules. The treated tissue was subsequently rinsed three times for 10 min each in phosphate-buffered saline (PBS) at room temperature (RT) and stored at 4 °C in PBS until use. Studies of commercially available porcine and bovine BHV have confirmed that approximately 95% α-Gal inactivation can be obtained by subjecting xenogeneic tissues to the FACTA procedure prior to GLUT treatment [126, 127]. In addition, eliminating the expression of antigens in transplanted biological tissues using gene editing techniques also provides an improved method to fight immune rejection [128].

#### 7.2.5 Polyphenol-Based Treatment

Phenolic compounds can exert potent anti-inflammatory effects by interfering with immune cell regulation, proinflammatory cytokine synthesis and gene expression [129]. In addition, polyphenols act to mask immunogenic epitopes and carboxyl residues involved in calcification through the formation of covalent and hydrogen bonds, which subsequently form stable complexes. Polyphenol-based treatment also improves the flexibility of the valve tissue, allowing for a more even distribution of mechanical stress across the leaflet surface and reducing the impact of mechanical stress on the valve through a more uniform and consistent valve switch. For commercial BHVs produced using different manufacturing methods, the application of polyphenol-based technologies in addition to other treatments can further improve their stabilizing properties [130]. Some researchers have also suggested that treatment with polyphenols alone could be potentially problematic, as calcium ions may bind to pericardium-bound polyphenols, which in turn can lead to calcification. Therefore, the investigators introduced ferric chloride, which reduced calcified deposits by competing with calcium ions through iron ions and better protected elastin [131].

#### 7.2.6 Innovative Biomaterial Treatment Techniques

In view of problems such as GLUT residue and calcification of traditional GLUT crosslinked BHV, non-GLUT crosslinked dry valve technology was developed. Dry biological valve technology is based on decellularization and decalcification through glycerolization, three-dimensional force-controlled drying, and ultralow-temperature vacuum lyophilization. The valve is dehydrated and dried and ultimately premounted, precut, and preloading, with a finer delivery system than traditional valves and with a tissue strength no less than that of similar products. In actual use, the valve can be used simply by rinsing with saline, which greatly decreases the loading time.

Linx AC anti-calcification technology is designed to minimize cholesterol uptake and stabilize leaflet collagen by extracting lipids and reducing free aldehyde groups, resulting in long-term performance and durability, with valves lasting 10–15 years or longer. A rabbit model study demonstrated less calcification in porcine valves treated with Linx AC technology compared with glutaraldehyde-treated controls [132]. A follow-up study revealed satisfactory long-term clinical outcomes and valve performance after implantation of the Epic Supra valve (treated with Linx AC technology) in the aortic position [133].

The ThermaFix process, developed in 2007, is a third-generation bioprosthetic valve anti-calcification technology that involves phospholipid extraction and an additional heat-treatment step that provides anti-calcification by covering the free aldehyde groups, removing most of the cholesterol and phospholipids in the leaflet, and stabilizing the leaflet collagen. This technology can also extend the valve life to 10–15 years or longer. In an animal experiment, ThermaFix process-treated bovine pericardium showed improved anti-calcification properties compared with those of conventional glutaraldehyde-treated controls [132]. Early results from a premarket clinical study in China suggest that the SAPIEN 3 valve, which is based on this anti-calcification technology, is safe and effective in Chinese patients undergoing transcatheter interventional valve replacement for high-risk aortic stenosis [134].

## 8. Imaging for Early Detection and Treatment Evaluation

Imaging is crucial for the early detection of calcification and for evaluating the response of prosthetic heart valves to anti-calcification therapies. Imaging techniques help clinicians identify calcific deposits at a stage when they are not clinically relevant. Therefore, patients can receive treatment before these deposits become a problem.

### 8.1 Echocardiography and Intraoperative Imaging

Echocardiography remains the gold standard for prosthetic valve imaging, as it provides real-time information on the function and structure of the valve. Procedures such as three-dimensional transesophageal echocardiography (3D TEE) allow for detailed imaging of the valve leaflets and calcifications [135, 136]. Doppler echocardiography quantifies flow across the valve and can identify any hemodynamic changes due to calcification [137]. Transesophageal and intracardiac echocardiography are used during the implantation of the valve to visualize the valve and check for early signs of calcification and proper positioning of the valve [138, 139]. Intraoperative imaging is very important for minimizing postoperative complications and for obtaining an instant assessment of the procedure’s effectiveness.

### 8.2 Computed Tomography (CT) Imaging for Calcification Detection

CT is a highly sensitive technique that provides detailed images of the calcification process and enables accurate measurement of calcified plaque, and it is also applied to assess calcification in aortic valves, providing an opportunity to quantify the calcification process and its dynamics in time [140]. Quantitative CT can monitor changes in calcification density, which can help in early diagnosis and evaluations of the efficacy of anti-calcification medications, including statins or phosphate binders [141]. CT imaging is also useful in preoperative evaluation, where surgeons can determine the degree of calcification in native and prosthetic valves. Recent developments in 3D reconstruction of CT images have enabled precise visualization of the valve morphology, which is essential for choosing the right type of valve and its placement during the operation [142, 143].

### 8.3 Positron Emission Tomography (PET) for Metabolic Activity Assessment

PET imaging, especially when integrated with CT (PET-CT), is helpful in evaluating metabolic activity related to early calcification. Here, radiotracers such as 18F-sodium fluoride (18F-NaF) are taken up in areas of active calcification and allow the clinician to identify early mineralization processes that are not visible on CT alone [144, 145, 146]. 18F-NaF PET imaging has high sensitivity for detecting microcalcifications and is a valuable tool in the assessment of early calcification in bioprosthetic valves [146, 147]. PET imaging is also used to assess the effectiveness of anti-calcification therapies, as decreased radiotracer uptake suggests decreased metabolic activity and possible calcification. Thus, PET imaging may assist clinicians in modifying treatment plans according to patients’ response to calcification in real time [148].

### 8.4 Magnetic Resonance Imaging (MRI) for Soft Tissue Characterization

MRI is generally less sensitive to calcific deposits compared with CT. However, it offers important information about the mechanical properties and materials used in prosthetic valves, helping to distinguish between calcified and noncalcified tissues. This information helps to understand the mechanical characteristics of the valve and identify potential zones of degeneration. T1 and T2 mapping are two of the most recent MRI techniques that can be used to quantify tissue stiffness, which is associated with early calcification and fibrosis [149, 150]. MRI is especially valuable in the assessment of tissue-engineered heart valves because it offers high soft tissue contrast and does not involve the use of ionizing radiation. This makes MRI suitable for use in pediatric and younger patients over long periods because frequent imaging does not contribute to the development of cancer due to radiation [151].

## 9. Conclusions

Prosthetic heart valves in clinical use are primarily mechanical or bioprosthetic, with BHV offering superior hemodynamics and reduced bleeding risks due to the absence of the need for anticoagulation therapy. However, calcification remains a significant limitation affecting BHV longevity. Local anti-calcification strategies are the main methods currently applied, primarily targeting the physical and chemical properties of valve materials. In the near future, advances in nanotechnology and tissue engineering could hold more promise for mitigating prosthetic heart valve calcification. Nonetheless, the transition from laboratory and animal studies to clinical applications has been limited. This gap highlights the need for a deeper understanding of calcification mechanisms and influencing factors in the human body, as well as the development of standardized evaluation criteria and more physiologically relevant models. Bridging this gap is crucial for selecting and advancing the most promising anti-calcification strategies for clinical use.
